# Anti-Adipogenic Effect of Secondary Metabolites Isolated from *Tetracera loureiri* on 3T3-L1 Adipocytes

**DOI:** 10.3390/ijms27031374

**Published:** 2026-01-29

**Authors:** Sung Ho Lim, Ju-Hyoung Park, Do-Hun Lee, Woo Young Bang, Jaeho Lee, Suon Sovann, Kry Masphal, Jae-Shin Kang, Dong-Wan Seo, Joa Sub Oh

**Affiliations:** 1 College of Pharmacy, Dankook University, Cheonan 31116, Republic of Korea; lamasungho@dankook.ac.kr (S.H.L.); jhp0607@dankook.ac.kr (J.-H.P.); ldh@dankook.ac.kr (D.-H.L.); 2 National Institute of Biological Resources, Environmental Research Complex, Incheon 22689, Republic of Korea; wybang@korea.kr (W.Y.B.); leejaeho@korea.kr (J.L.); diatomlover@gmail.com (J.-S.K.); 3 Forestry and Fisheries, Forestry Administration, Ministry of Agriculture, Phnom Penh 120801, Cambodia; suonsovannkh@yahoo.com (S.S.); maffa162@gmail.com (K.M.)

**Keywords:** *Tetracera loureiri*, triterpenoids, flavonoids, simple phenols, 3T3-L1 adipocytes

## Abstract

A species of the genus *Tetracera* has been used as herbal medicine in traditional Indian *Tetracera loureiri* medicine. *Tetracera loureiri*, a plant from the Dilleniaceae family is considered one of the most valuable herbs in Thailand and is native to Southeast Asia. However, the anti-obesity effects of *Tetracera loureiri* extract have not been reported. In this study, we screened the effect of EtOH extract on lipid accumulation in a 3T3-L1 adipocyte model at various concentrations using Oil Red O staining, and the results were visualized under a light microscope. The fractionation of the soluble CH_2_Cl_2_ and EtOAc fractions from the EtOH extract revealed that both fractions significantly inhibited lipid accumulation in adipocytes at 2.5, 5, and 10 μg/mL, compared to *n*-hexane, *n*-BuOH, and aqueous extracts. Bioactivity-guided fractionation of the CH_2_Cl_2_ and EtOAc extract led to the phytochemical investigation of 10 secondary metabolites (1–10), and the structure of these compounds was identified using various spectroscopic methods. All isolated compounds were evaluated for their ability to inhibit adipogenesis at a concentration of 2.5, 5, and 10 μM compared with positive control (Orlistat 100 μg/mL); in particular, compounds 1–3, 5, and 7–8 showed 57.39 ± 6.98, 19.35 ± 4.53%, 75.81 ± 1.75%, 17.61 ± 1.62%, 19.83 ± 5.27 and 14.66 ± 3.85% reduction in fat accumulation at 10 μMm in 3T3-L1 adipocytes, respectively. The activity of these compounds also inhibited the secretion of adiponectin and leptin in 3T3-L1 adipocytes, suggesting their role in disrupting adipocyte function and metabolic regulation. Therefore, the results herein provide experimental evidence supporting the potential of *Tetracera loureiri* extracts as an anti-obesity therapeutic agent.

## 1. Introduction

Obesity is a chronic and multifaceted condition characterized by excessive fat accumulation that poses risks to overall health. This condition is a disease caused by a combination of various factors which lead to expansion of adipose tissue, and it is recognized as a serious problem, because obesity can affect skeletal muscle (lipid accumulation and peripheral insulin resistance), cardiac muscle (lipid deposition), and endothelial dysfunction [1]. Effective management typically involves a comprehensive approach combining lifestyle modifications, such as diet and exercise, with pharmacological interventions targeting appetite regulation, nutrient absorption, and metabolism [2,3]. In this context, evaluating bioactive compounds that influence adipocyte function is crucial for identifying potential therapeutic agents. The 3T3-L1 cell line is widely used to study adipocyte differentiation and lipid accumulation. In this study, natural extracts and their specific components were evaluated for their effects on adipocyte differentiation and fat storage, exploring their potential as anti-obesity agents.

*Tetracera loureiri* (*T. loureiri*) is a woody climber in the Dilleniaceae family and an angiosperm native to deciduous or evergreen forests in Southeast Asia. In traditional Indian medicine, some species of this genus are considered valuable herbs for treating dysentery, hepatitis, blennorrhagia, and febrifuge; as diuretic agents; for alleviation of fatigue; and for treatment of jaundice [4]. *T. loureiri* extract has been reported to possess potential therapeutic value in some liver disorders and as an anti-inflammation agent [5,6,7,8] due to the presence of acylated triterpenoids, flavonoids, and lignans in the stems [9]. These compounds have exhibited biological activities such as anti-cancer, anti-atherogenic, anti-HIV, and anti-leishmanial [10,11,12,13]. Despite these pharmacological investigations, there has been no previous report on the potential of *T. loureiri* in inhibiting adipocyte differentiation or addressing obesity-related metabolic issues. *T. loureiri*, with its rich profile of secondary metabolites, offers a promising opportunity to identify novel compounds that could target obesity-related metabolic disorders. This study not only aims to uncover previously unreported biological activities of its isolated compounds but also seeks to contribute to the broader understanding of plant-based interventions for metabolic conditions associated with obesity. Accordingly, we investigated *T. loureiri* to reveal its secondary metabolites and their biological activities on a murine 3T3-L1 preadipocyte cell line using Oil Red O assays. Effects on lipid accumulation in mature adipocytes were assessed as a measure of potential anti-obesity activity for initial screening in vitro rather than as a definitive demonstration of efficacy in vivo. Bioactivity-guided fractionation of the whole-plant extract (70% EtOH) led to isolation of three triterpenoids (maslinic acid, corosolic acid, and eucalyptolic acid), four flavonoids (quercetin, quercitrin, kaempferol, and rhamnocitrin) and three simple phenolics (gallic acid, ethyl gallate, and *p*-coumaric acid). The structure of the known compounds was characterized by using spectral experiments (1D/2D NMR and HR-MS). These compounds have been extensively reported for their effects on adipocyte differentiation or metabolic functions [14,15,16,17,18,19,20], except for eucalyptolic acid. Furthermore, we evaluated the effects of the isolated compounds on adipocyte differentiation and the expression of metabolic markers such as adiponectin and leptin, which are important indicators of metabolic function [21,22].

## 2. Results

### 2.1. Identification of Isolated Compounds from T. loureiri Extracts

**Figure 1:**
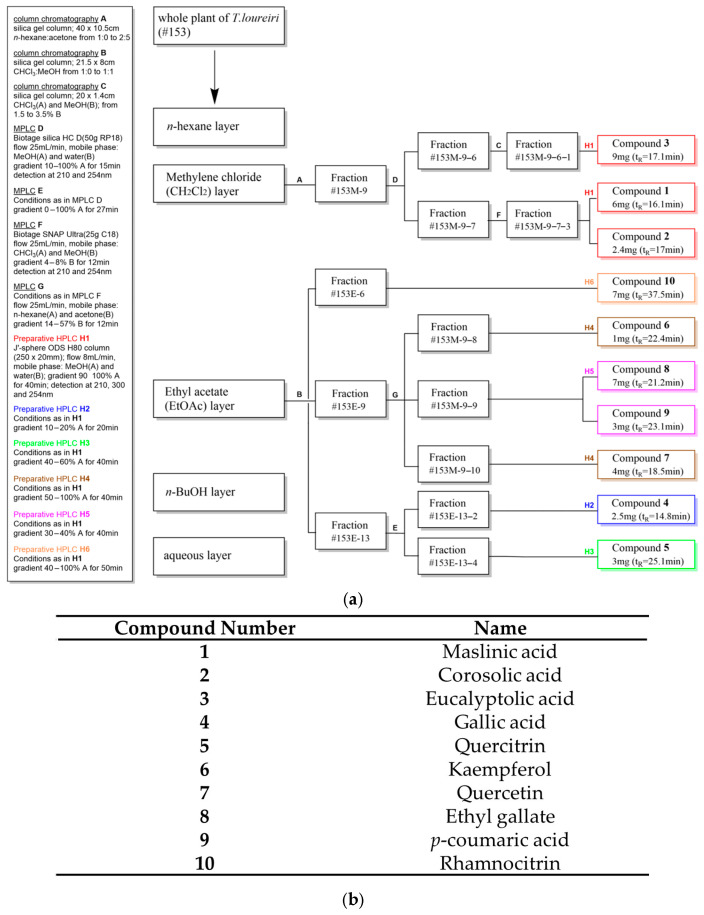
The isolation of compounds from *T. loureiri*. The isolation scheme of compounds 1–10; The isolation scheme of compounds 1-10 and colors indicate different prep HPLC methods (**a**). Three triterpenoids, four flavonoids, and three simple phenolics were isolated from *T. loureiri*. These include maslinic, corosolic, and eucalyptolic acids; quercetin, quercitrin, kaempferol, and rhamnocitrin; and gallic acid, ethyl gallate, and *p*-coumaric acid (**b**).

**Figure 2:**
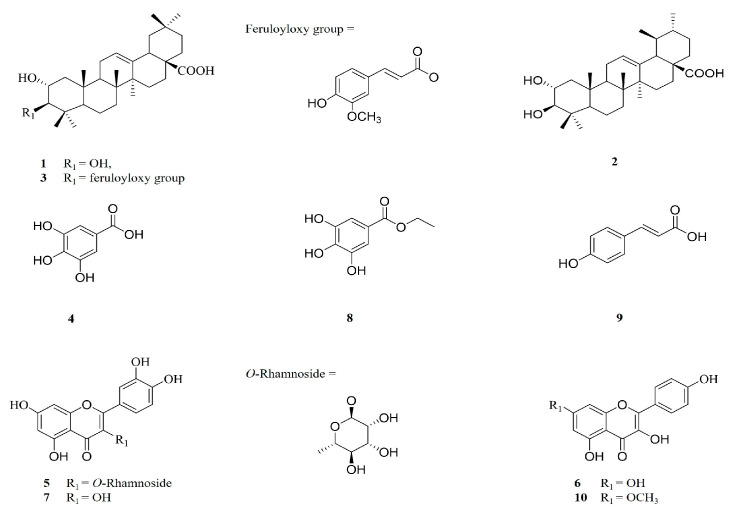
Chemical structures of compounds 1–10 from *T. loureiri*. 1: Maslinic acid; 2: corosolic acid; 3: eucalyptolic acid; 4: gallic acid; 5: quercitrin; 6: kaempferol; 7: quercetin; 8: ethyl gallate; 9: *p*-coumaric acid; 10: rhamnocitrin.

**Figure 3:**
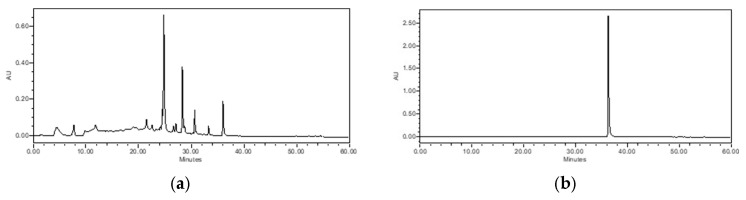
Chromatogram of indicator substance from *T. loureiri* for the EtOAc layer of *T. loureiri* extract and chemical chromatogram identified by high-performance liquid chromatography (HPLC) at 360nm: (**a**) EtOAc fractionation extract from *T. loureiri* and (**b**) chromatogram of rhamnocitrin.

The compounds isolated from the CH_2_Cl_2_ (1–3) and EtOAc layer (4–10) of *T. loureiri* were identified through NMR spectroscopic techniques (Figure 1 and Figure 2 and Supplementary Table S1). NMR data were identified by comparison with reported values in the literature [23,24,25]. One of the isolated compounds, rhamnocitrin, was designated as a marker compound based on its availability, repeatability, and reproducibility as indicated by HPLC chromatogram (Figure 3).

### 2.2. Effects of T. loureiri on Cell Viability in 3T3-L1 Cells

**Figure 4:**
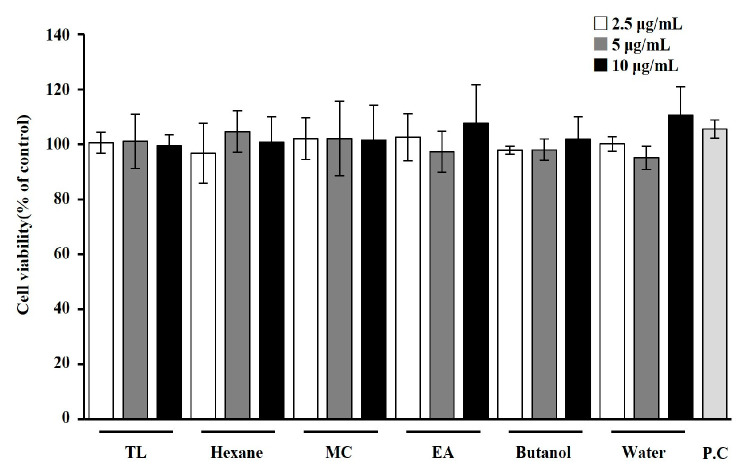
Effects of solvent extracts from *T. loureiri* on 3T3-L1 cell viability. Cell viability was assessed following the procedure described in Section 4; 3T3-L1 cells were treated with the sample for 24 h. The results from at least three independent experiments (mean ± SD) are shown as a percentage of the untreated control. TL: *Tetracera loureiri*; MC: methylene chloride layer; EA: ethyl acetate layer; P.C: Orlistat.

**Figure 5:**
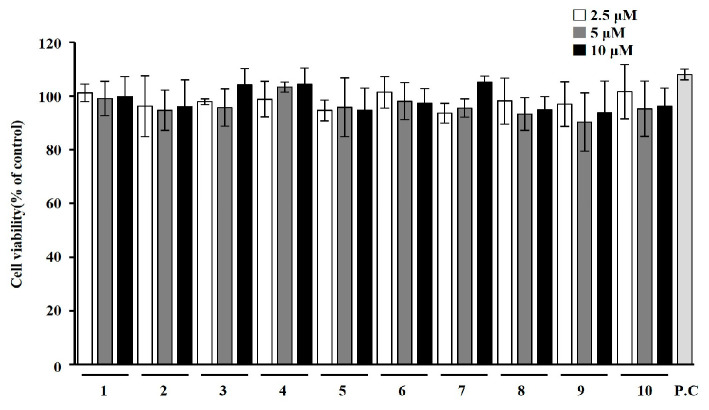
Effects of chemicals *T. loureiri* on 3T3-L1 cell viability. Cell viability was assessed following the procedure described in Section 4. The 3T3-L1 cells were treated with the sample for 24 h: The results from at least three independent experiments (mean ± SD) are shown as a percentage of the untreated control. 1: Maslinic acid; 2: corosolic acid; 3: eucalyptolic acid; 4: gallic acid; 5: quercitrin; 6: kaempferol; 7: quercetin; 8: ethyl gallate; 9: *p*-coumaric acid; 10: rhamnocitrin; P.C: Orlistat.

We first analyzed the effect of *T. loureiri* on cell viability in 3T3-L1 cells. As shown in Figure 4 and Figure 5, treatment with various solvent extracts and compounds from *T. loureiri* showed no toxicity at concentrations lower than 10 μg/mL or 10 μM. Based on these findings, subsequent experiments were conducted to examine the effects of the compound from *T. loureiri* at a concentration of 10 μM, with Orlistat (100 μg/mL), a widely used anti-obesity drug, used as the positive control (P.C).

### 2.3. Effects of T. loureiri on Lipid Accumulation in 3T3-L1 Cells

**Figure 6:**
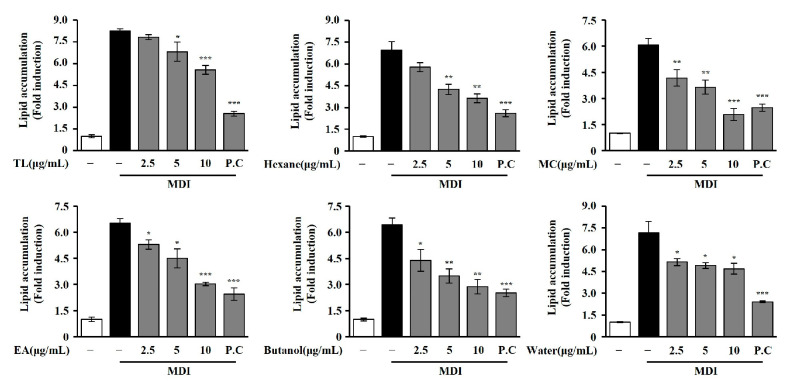
Effect of solvent extracts from *T. loureiri* on the inhibition of lipid accumulation during adipocyte differentiation. The 3T3-L1 cells were differentiated over 7 days. Lipid accumulation assays were performed as described in Section 4. The results from at least three independent experiments (mean ± SD) are shown as a percentage of the untreated control. TL: *Tetracera loureiri*; MC: methylene chloride layer; EA: ethyl acetate layer; P.C: Orlistat; MDI: 3-isobutyl-1-methylxanthine, dexamethasone, and insulin. Statistical significance is indicated as follows: * p < 0.05, ** p < 0.01, *** p < 0.001 compared to untreated cells.

The ethanol extract of *T. loureiri* suppressed lipid accumulation in 3T3-L1 adipocytes in a concentration-dependent manner, with effects observed at concentrations of 2.5, 5, and 10 μg/mL. To investigate the anti-adipogenic constituents in ethanol extract, sequential extraction was performed with *n*-hexane, CH_2_Cl_2_, EtOAc and *n*-BuOH. These fractionations were tested for anti-adipogenic effects and their inhibitory effects on lipid accumulation in 3T3-L1 cells at 2.5, 5, and 10 μg/mL (Figure 6). All solvent extracts were conducted at the same concentrations to compare relative activity with Orlistat (100 μg/mL) used as the P.C. In our reports, the *n*-hexane, CH_2_Cl_2_, EtOAc, and *n*-BuOH extracts revealed significantly concentration-dependent anti-adipogenic effects at the highest concentration test of 10 μg/mL.

### 2.4. Effects of Chemicals from T. loureiri on Inhibition of Lipid Accumulation in 3T3-L1 Adipocytes

**Figure 7:**
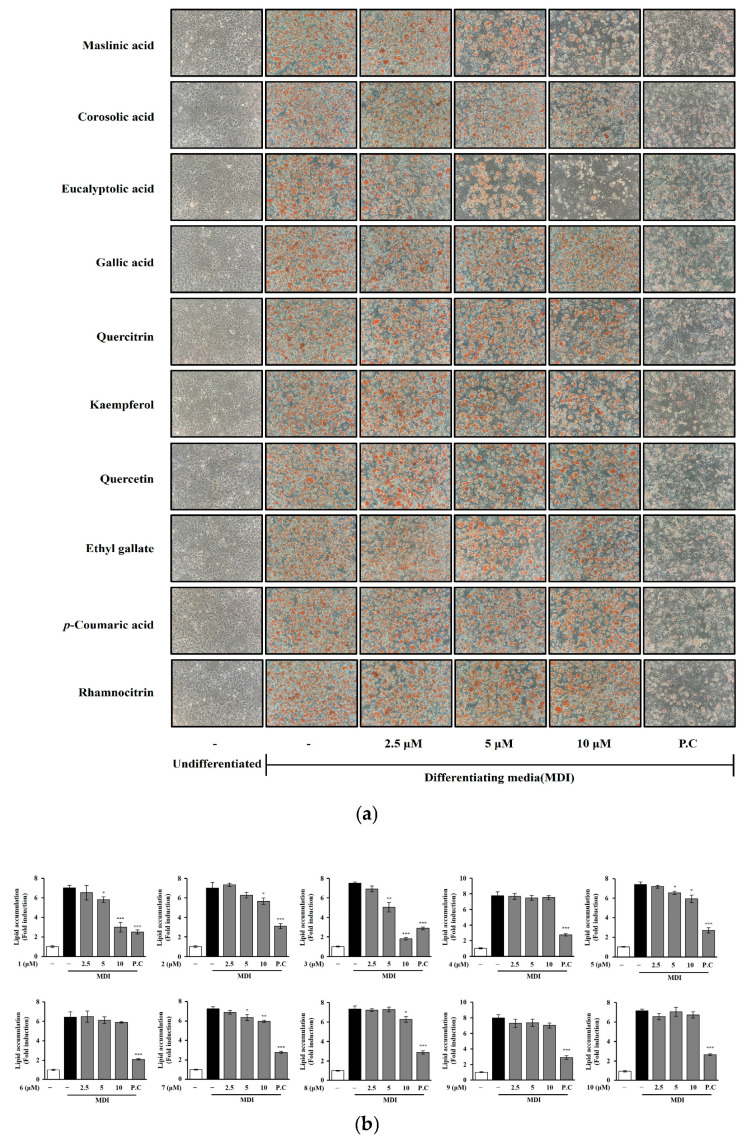
Effect of chemicals from *T. loureiri* on the inhibition of lipid accumulation during adipocyte differentiation. 3T3-L1 cells were differentiated over 7 days: (**a**) ORO staining and (**b**) lipid accumulation assays were performed as described in Section 4. The results from at least three independent experiments (mean ± SD) are shown as a percentage of the untreated control. 1: Maslinic acid; 2: corosolic acid; 3: eucalyptolic acid; 4: gallic acid; 5: quercitrin; 6: kaempferol; 7: quercetin; 8: ethyl gallate; 9: *p*-coumaric acid; 10: rhamnocitrin; P.C: Orlistat; MDI: 3-isobutyl-1-methylxanthine, dexamethasone, and insulin. Statistical significance is indicated as follows: * p < 0.05, ** p < 0.01, *** p < 0.001 compared to untreated cells.

The isolated compounds were obtained from the CH_2_Cl_2_ and EtOAc extracts of *T. loureiri*. Three triterpenoids were isolated from the CH_2_Cl_2_ extract, and four flavonoids and three simple phenolics were isolated from the EtOAc extract. The anti-adipogenic effects of these compounds (1–10) were evaluated at 2.5, 5, and 10 μM concentrations on 3T3-L1 preadipocytes, and their effects were visualized under a light microscope (Figure 7a). Three triterpenoids (maslinic acid (1), corosolic acid (2), and eucalyptolic acid (3)) were the most active in terms of adipose lipid inhibition. The two flavonoids quercitrin (5) and quercetin (7) showed slight activity. One simple compound, phenolic ethyl gallate (8), revealed an inhibitory effect on lipid accumulation starting from a concentration of 10 μM. Out of six compounds, two flavonoids (kaempferol (6) and rhamnocitrin (10)) and two simple phenolic compounds (gallic acid (4) and *p*-coumaric acid (9)) were inactive. This information is detailed in Figure 7b and Supplementary Table S2.

### 2.5. Effects of Chemicals from T. loureiri on Adiponectin and Leptin Secretion in 3T3-L1 Adipocytes

**Figure 8:**
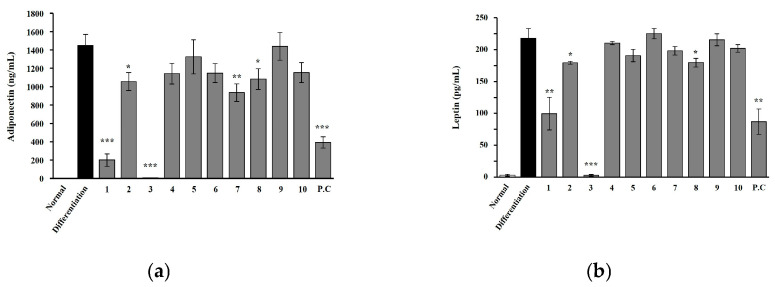
Effect of *T. loureiri* on the secretion of adiponectin and leptin during adipocyte differentiation. The 3T3-L1 cells were differentiated over 7 days, and the ELISA was performed to assess the effects of TL on (**a**) adiponectin and (**b**) leptin secretion. The results from at least three independent experiments (mean ± SD) are shown as a percentage of the untreated control. 1: Maslinic acid; 2: corosolic acid; 3: eucalyptolic acid; 4: gallic acid; 5: quercitrin; 6: kaempferol; 7: quercetin; 8: ethyl gallate; 9: *p*-coumaric acid; 10: rhamnocitrin; P.C: Orlistat. Statistical significance is indicated as follows: * p < 0.05, ** p < 0.01, *** p < 0.001 compared to untreated cells.

To investigate peptides secreted by adipocytes, we further evaluated the quantity of adiponectin and leptin in 3T3-L1 cells, measuring the expression levels of related compounds at 10 μM. Among the 10 isolated compounds, the triterpenoids maslinic acid and eucalyptolic acid notably reduced adiponectin and leptin levels in 3T3-L1 cells. This information is presented in Figure 8 and Supplementary Table S3.

## 3. Discussion

In the initial study, the 70% ethanol extract of *T. loureiri* showed anti-adipogenic effects according to Oil Red O staining and toxicity measured by MTT assay in 3T3-L1 cells treated up to 10 μg/mL. The crude extracts and solvent fractions of *T. loureiri* were evaluated for their inhibitory effects on adipocyte differentiation at the same concentration (2.5, 5, and 10 μg/mL). The *n*-hexane, CH_2_Cl_2_, EtOAc, *n*-BuOH and aqueous extracts of *T. loureiri* reduced lipid accumulation by percentages of 47.65 ± 4.32, 65.73 ± 5.50, 53.53 ± 1.51, 55.12 ± 6.42 and 34.73 ± 5.23%, respectively. Therefore, to assess the effects of isolated phytochemicals on 3T3-L1 preadipocytes, additional isolation steps were performed using CH_2_Cl_2_ and EtOAc extracts. The purified compounds were then applied to adipocytes at concentrations ranging from 2.5 to 10 μM compared with Orlistat. Although Orlistat exerts anti-obesity action primarily by inhibiting lipid absorption in the intestine, we show here that it also inhibits 3T3-L1 adipocyte differentiation in vitro, which qualified it as a positive control for our study. In this study, Orlistat was used as a positive control to provide a benchmark for overall anti-obesity effects, while the focus remains on modulating adipocyte differentiation. To assess the potential anti-obesity activity in an initial screening model in vitro, intracellular lipid accumulation in differentiated adipocytes was quantified using Oil Red O staining [26,27]. Furthermore, leptin and adiponectin are markers of adipocyte differentiation but are more crucial for assessing adipocyte function and metabolic status [28,29]. We assessed the inhibition of adipocyte differentiation not only by Oil Red O staining but also by measuring the levels of adiponectin and leptin. As shown in Figure 7, the isolated triterpenoids, along with several flavonoids and a simple phenol from *T. loureiri,* exhibited a tendency to reduce lipid accumulation. Total 10 isolated compounds were classified into triterpenoid, flavonoid, and simple phenols, as shown in Figure 2. As shown in Figure 1, the three pentacyclic triterpenes (1–3) known as maslinic acid (2α, 3β-dihydroxyolean-12-en-28-oic acid) (1), corosolic acid (2α,3β-2,3-dihydroxyurs-12-en-28-oic acid) (2), and eucalyptolic acid (2α-hydroxy-3β-E-feruloyloxy-olean-12-en-28-oic acid) (3) were contained in the CH_2_Cl_2_ extract. Isolated triterpenoids of oleanane type have a hydroxy group at position 2 and 3 in ring A with a carboxyl group at position 17 between rings D and E. Among them, the isolated maslinic acid shows a *cis* configuration, while corosolic acid adopts a trans configuration in the stereochemistry of the ring E [30,31]. We found that these two similar pentacyclic triterpenoids reduced lipids in adipocytes by 57.39 ± 6.98 and 19.35 ± 4.53%, respectively, up to a high concentration, which was confirmed to be a value considered to significantly inhibit lipid accumulation in adipocytes, as shown in Figure 7 and Figure 8. Maslinic acid and corosolic acid have been demonstrated as potential ani-adipogenic compounds in 3T3-L1 by regulating several molecular and transcription factors such as peroxisome proliferator-activated receptor γ (PPARγ) and adipocyte fatty acid-binding protein (aP2) and intracellular Ca2^+^ levels [14,32]. Furthermore, based on the molecular structure of maslinic acid, eucalyptolic acid—which has a β-oriented feruloyl oxy group at 3 in ring A [33]—was shown to significantly reduce the accumulation of lipids and release of adipokines in 3T3-L1 adipocytes, as shown in Figure 7 and Figure 8. As shown in Figure 1 and Figure 2, all flavonoids of *T. loureiri* were isolated from the EtOAc extract, and these were subclassified as flavonols which consisted of 2-phenyl-1,4-benzopyrone with several hydroxyl groups [34] known as gallic acid (3,4,5-trihydroxybenzoic acid) (4), quercitrin (5), kaempferol (3,5,7-trihydroxyflavone) (6), Quercetin (7), ethyl gallate (3,4,5-trihydroxybenzoic acid-ethyl ester) (8), and *p*-coumaric acid (4-hydroxycinnamic acid) (9), and rhamnocitrin (kaempferol 7-*O*-methyl ether) (10). Quercitrin (5) and quercetin (7) have been extensively reported to modulate regulation of pathways related to both adipogenesis and lipolysis, such as the AMPK, PI3K/Akt and SIRT1 signaling pathways [35,36,37]. As shown in Figure 7 and Figure 8, quercetin (7), also known as 2-(3,4-dihydroxyphenyl)-3,5,7-trihydroxy-4H-chromen-4-one, inhibited accumulation of lipids and release of adiponectin. The chemically substituted form of quercetin’s closed pyran ring to rhamnoside (quercetin-O-rhamnoside) [38], quercitrin (5), reduced lipid content in adipocytes. Isolated kaempferol (3,5,7-trihydroxyflavone) (6) is the form of quercetin with the removed 3-hydroxy group [39] in ring B, and rhamnocitrin (kaempferol 7-*O*-methyl ether) (10) is the form of kaempferol with ring A replaced by a methoxy substituent at position 7 [40]. These two flavonols showed the effect of inhibiting lipids in adipocytes by 8.22 ± 1.32% and 5.98 ± 4.53% even at a high concentration of 10 μM, as shown in Figure 7. This means it is difficult to consider them anti-adipocyte components, as confirmed in our study. The isolated simple phenolic compounds that share a common aromatic ring include gallic acid (3,4,5-trihydroxybenzoic acid) (4), ethyl gallate (3,4,5-trihydroxybenzoic acid-ethyl ester) (8), and *p*-coumaric acid (4-hydroxycinnamic acid) (9). Gallic acid and ethyl gallate contain three hydroxyl group, while *p*-coumaric acid features a single hydroxyl and a propenoic acid side chain [41]. Ethyl gallate, an esterified derivative of gallic acid, replaces the carboxyl with an ethyl ester. Ethyl gallate is a commonly abundant plant phenolic compound that upregulates glucose uptake and downregulates adipogenesis in 3T3-L1 cells by inhibiting PTPN6, demonstrating its potential as a dual-targeting (PTPN6 and PPARγ) agent for improving insulin sensitivity and reducing lipid accumulation [42]. We found that ethyl gallate was moderately active in inhibiting lipid accumulation, and other phenolic compounds were negligibly active in suppressing lipid accumulation in adipocytes, as shown in Figure 7. Adiponectin and leptin are established markers of adipocyte differentiation, reflecting both the maturation and metabolic activity of adipocytes. Adiponectin, predominantly secreted by mature adipocytes, is known to enhance insulin sensitivity and exert anti-inflammatory effects, reflecting the functional maturation of adipocytes. Leptin, whose secretion increases with adipocyte differentiation, plays a key role in energy homeostasis and serves as a marker of cellular metabolic status [43,44]. As shown in Figure 8, the observed changes in adiponectin and leptin levels should be interpreted as effects related to altered adipocyte differentiation at the cellular level, rather than as indicators of systemic energy balance. The compounds exhibiting inhibitory effects on adipocyte differentiation showed a significant correlation with the expression of hormones post-differentiation, suggesting that they may have affected the metabolic functions of adipocytes such as insulin sensitivity and energy expenditure [45]. Given that adipocyte differentiation is regulated by complex biochemical networks, the concentrations used in this study were selected to elucidate potential biological activity and underlying mechanisms. However, extrapolation of these findings to in vivo conditions should be approached with caution, as achievable concentrations are influenced by pharmacokinetic factors. Further in vivo and pharmacokinetic studies are required to validate the physiological relevance of these observations. Although inhibition of adipocyte differentiation in 3T3-L1 cells is frequently used as an initial screening model for anti-obesity candidates, such effects cannot be directly translated into beneficial metabolic outcomes in vivo [46,47]. Indeed, impaired adipogenesis may under certain conditions lead to abnormal lipid accumulation and metabolic dysfunction. Therefore, the present findings should be interpreted as preliminary mechanistic observations, and further in vivo studies are required to clarify the metabolic consequences of the observed effects. The observed changes in adiponectin and leptin levels in response to treatment indicate that the compounds not only inhibit lipid accumulation but also affect adipocyte functional maturation. Based on these findings, the triterpenoids (maslinic acid, corosolic acid, and eucalyptolic acid), flavonoids (quercetin, quercitrin), and simple phenol (ethyl gallate) isolated from *T. loureiri* demonstrated potential anti-obesity effects. This was evidenced by their dose-dependent inhibition of adipocyte differentiation and lipid accumulation in 3T3-L1. In this study, we explored the effects of naturally derived compounds on adipocyte differentiation in a 3T3-L1 preadipocyte model. The primary goal of using Oil Red O staining and adipokine analysis in vitro does not provide definitive anti-obesity efficacy in vivo, but it serves as an initial screening of potential anti-obesity compounds. These results indicate that several compounds from *T. loureiri* can inhibit adipocyte differentiation and lipid accumulation in vitro. However, it is important to emphasize that such in vitro findings do not directly translate into anti-obesity effects or clinical benefits in vivo. While inhibition of adipocyte differentiation may reduce fat accumulation in a cell model, adipogenesis in living organisms is regulated by complex physiological systems, and disruption of these processes may not produce comparable effects in vivo. Therefore, the present findings should be interpreted as preliminary mechanistic evidence, and further in vivo and pharmacokinetic studies are required to evaluate the physiological relevance and therapeutic potential of these compounds.

## 4. Materials and Methods

### 4.1. Plant Materials

*T. loureiri* whole plants were collected from the Forestry Administration of Cambodia. Botanical identification of *T. loureiri* was performed by one of the authors, Dr. J. S. Kang, and its certificate specimen was deposited at the National Institute of Biological Resources, Republic of Korea. Dried whole plants of *T. loureiri* (1 kg) were pulverized and extracted with 70% ethanol at room temperature for 3 days. The extract was filtered and concentrated for complete evaporation of the ethanol using vacuum evaporation. The extract was completely freeze-dried to obtain a powder.

### 4.2. General Experimental Procedures

Medium-pressure liquid chromatography (MPLC) (Biotage^®^ Isolera, Uppsala, Sweden) and open column chromatography were used as instruments for the isolation of substances. The extract fractionations were concentrated using a rotary evaporator (EYELA, Tokyo, Japan) to completely evaporate the remaining aqueous solution. The target compounds were purified by using high-performance liquid chromatography [Waters HPLC, consisted of 2998 (PDA), 2707 (Auto sampler), 1525 (pump), Prep degasser] using DI water, MeOH, and acetonitrile from Burdick & Jackson™ (Honeywell, Muskegon, MI, USA). Analysis was performed on YMC PACK ODS A (12 μm, S-5 μm, 250 × 4.6 mm, YMC, Kyoto, Japan) and preparative separation on a J’-sphere ODS-H80 (8 μm, S-4 μm, 250 × 20 mm, YMC Co., Ltd., Kyoto, Japan). All data acquisition and analysis were controlled using Empower 3 software (Waters corporation, Milford, MA, USA). TLC was performed on silica gel 60 F254 plates (Merck KGaA, Darmstadt, Germany) and silica gel 60 RP-18 F254s plates (Merck KGaA, Darmstadt, Germany) with UV (254 and 365 nm) and spraying vanillin sulfuric acid reagent (3 min heating) used to visualize spots. The 1H-nuclear magnetic resonance (NMR; 700 MHz), 13C-NMR (175 MHz) spectra were obtained using a Bruker Ascend III NMR spectrometer in CDCl_3_ and MeOH. The ESI-MS spectra measurement of the isolated compounds were obtained using an Agilent 6130 series quadrupole LC/MS system (Agilent Technologies, Santa Clara, CA, USA).

### 4.3. Isolation of Chemicals from the Active Fractions of T. loureiri

The whole plants of *T. loureiri* were extracted with 70% aqueous EtOH. The crude extract (1.4 kg) was partitioned with *n*-hexane (5 L), CH_2_Cl_2_ (5 L), EtOAc (5 L), and *n*-BuOH (2.5 L, *n*-BuOH/DI water; 7:3), yielding 28 g, 225 g, 64 g, and 280 g, respectively. Among the layers obtained, the CH_2_Cl_2_ layer exhibited the highest anti-obesity activity on 3T3-L1 and was selected for further bioactivity-guided fractionation. The CH_2_Cl_2_ layer was subjected to open column chromatography (10.5 × 40 cm) under a gradient elution of *n*-hexane (solvent A) and acetone (solvent B) [1:0 (A:B) to 2:5 (A:B)] to yield 14 fractions (#153M-1~14). The Oil Red O assays revealed the most potent fractions to be #153M-9 (5.5 g). Accordingly, #153M-9 was eluted with 10% aqueous MeOH to pure MeOH from MPLC to obtain 7 subfractions (#153M-9-1~7). Fraction #153M-9-7 was subjected to MPLC using a solvent of 4–8% gradient MeOH in CHCl_3_ to obtain 4 fractions (#153M-9-7-1 − #153M-9-7-4). Subfraction #153M-9-7-3 (52 mg) was purified by reverse-phase (MeOH/DI water: 90:10–100:0, UV: 210 nm, flow rate: 8 mL/min, 8 μm, 250 × 20 mm) prep HPLC to afford compound 1 (6 mg) and compound 2 (2.4 mg). Bioactive fraction #153M-9-6 (388 mg) was further separated using open column chromatography (1.4 × 20 cm) using normal-phase (silica gel, 70–230 mesh) with different ratios of the CHCl3 and MeOH in the volume of 170 mL to yield 4 subfractions (#153M-9-6-1–#153M-9-6-4) based on thin-layer chromatography (TLC). Subfraction #153M-9-6-1 (38 mg) was further purified by prep HPLC using reverse-phase (MeOH/DI water: 90:10–100:0, UV: 210 nm, flow rate: 8 mL/min, 8 μm, 250 × 20 mm) to afford compound 3 (9 mg). The ethyl acetate layer was subjected to open column chromatography (8 × 21.5 cm) with gradient 0% MeOH in CHCl_3_ to 50% MeOH to yield 15 fractions (#153E-1–#153E-15). Among them, #153E-13 (11 g) was separated to MPLC using Biotage^®^ sfär silica HC D (50 g, 20 μm particle size) with MeOH gradient solution (0–100% MeOH, 27 min) and obtained 4 subfractions (#153E-13-1–#153E-13-4). Thin-layer chromatography (TLC) was used to create a chemical profile of the subfractions at UV (wavelength: 254 and 365 nm) and vanillin sulfuric acid reagent followed by heating for 3 min. Subfraction 20 mg of #153E-13-2 (458 mg) was purified by reverse-phase (MeOH/DI water: 10:90–20:80, UV: 210–300 nm, flow rate: 8 mL/min, 8 μm, 250 × 20 mm) prep HPLC to obtain compound 4 (2.5 mg). Subfraction 106 mg of #153E-13-4 (6.8 g) was purified by reverse-phase (MeOH/DI water: 40:60–60:40, UV: 210–300 nm, flow rate: 8 mL/min, 8 μm, 250 × 20 mm) prep HPLC to obtain compound 5 (3 mg). Similarly, fraction #153E-9 (1 g) was processed using MPLC with a C18 column (SNAP Ultra 25 g) using a gradient solvent of 14% acetone in *n*-hexane to 57% to yield 12 subfractions (#153E-9-1−#153E-9-12). Among them, subfraction #153E-9-8 (46 mg) was purified by reverse-phase (MeOH/DI water: 50:50–100:0, UV: 210–300 nm, flow rate: 8 mL/min, 8 μm, 250 × 20 mm) prep HPLC to obtain compound 6 (1 mg). Subfraction 20 mg of #153E-9-10 (263 mg) was purified by reverse-phase column (MeOH/DI water: 50:50–100:0, UV: 210–300 nm, flow rate: 8 mL/min, 8 μm, 250 × 20 mm) prep HPLC to obtain compound 7 (4 mg). Subfraction 105 mg of #153E-9-9 (211 mg) was purified by reverse-phase (MeOH/DI water: 30:70-40:60, UV: 210–300 nm, flow rate: 8 mL/min, 8 μm, 250 × 20 mm) prep HPLC to obtain compound 8 (7 mg) and compound 9 (3 mg). In addition, the fraction 40 mg of #153E-6 (1.4 g) was purified by reverse-phase (MeOH/DI water: 40:60–90:10, UV: 210–300 nm, flow rate: 8 mL/min, 8 μm, 250 × 20 mm) prep HPLC to obtain compound 10 (7 mg). The isolation process employed in the present study is outlined in Figure 1.

### 4.4. Cell Culture and Differentiation Conditions

The 3T3-L1 cells were purchased from the American Type Culture Collection (ATCC, Manassas, VA, USA) and cultured in 3T3-L1 preadipocyte medium (PM, ZenBio, Durham, NC, USA). On day 4, to induce adipocyte differentiation, 3T3-L1 cells were seeded at a density of 5 × 10^4^ cells/well in 24-well plates and incubated for 4 days. After 4 days, cells were switched to adipocyte differentiation medium (DM, ZenBio, Durham, NC, USA), followed by adipocyte maintenance medium (AM, ZenBio, Durham, NC, USA) on day 3 and 5. The differentiation of 3T3-L1 cells was terminated on day 7.

### 4.5. Cell Viability Assay

To assess cell viability, a 3-(4,5-dimethylthiazol-2-yl)-2,5-diphenyltetrazolium bromide (MTT) assay was performed. The 3T3-L1 cells were seeded at a density of 5 × 10^3^ cells/well in 96-well plates and incubated for 24 h. After 24 h, the cells were treated with the indicated concentrations of each sample. Following 24 h of treatment, the supernatant was removed, and 100 μL of MTT solution (0.5 mg/mL, Duchefa Biochemie, Haarlem, The Netherlands) was added to each well. After 4 h of incubation, the supernatants were aspirated, and 100 μL of dimethyl sulfoxide (Duchefa biochemie, Haarlem, The Netherlands) was added to each well. The absorbance was measured at 540 nm using a SpectraMAX 190 microplate reader (Molecular devices, Sunnyvale, CA, USA).

### 4.6. Oil Red O Assays

On day 7 of adipocyte differentiation, 3T3-L1 cells were washed twice with PBS and fixed in 4% formaldehyde solution for 30 min at room temperature. The cells were then stained with Oil Red O (ORO, Sigma-Aldrich, St. Louis, MO, USA) solution. After washing twice with ethanol and twice with distilled water, the cells were observed at 200 magnification using a CKX 53 light microscope (Olympus, Tokyo, Japan). For quantitative analysis, the stained lipid droplets were dissolved in isopropanol with 4% NP-40 (Sigma-Aldrich, St. Louis, MO, USA), and the absorbance was measured at 510 nm using a SpectraMAX 190 microplate reader.

### 4.7. Enzyme-Linked Immunosorbent Assay (ELISA)

The concentration of adiponectin and leptin was determined using ELISA kits (Adiponectin: Merck, Darmstadt, Germany; leptin: R&D systems, Minneapolis, MN, USA). On day 7 of adipocyte differentiation, cell supernatants were harvested, and an ELISA assay was performed according to the manufacturer’s instructions. The absorbance was measured at 450 nm using a SpectraMAX 190 microplate reader.

### 4.8. Statistical Analysis

Statistical analysis was performed using Student’s *t*-test, with results derived from three independent experiments. Statistical significance was considered at * p < 0.05, ** p < 0.01 and *** p < 0.001.

## 5. Conclusions

As part of a project to discover biologically active natural products, this study aimed to identify anti-adipogenic secondary metabolites from whole-plant 70% EtOH extract of *T. loureiri* that inhibits lipid accumulation of 3T3-L1 cells. The 70% EtOH extract of *T. loureiri* displayed significant anti-adipogenic effects on 3T3-L1 adipocytes according to Oil Red O assays, and their constituents were identified as several triterpenoids, flavonoids, and simple phenols. The CH_2_Cl_2_ and EtOAc fractions were prepared from the initial ethanol extract of *T. loureiri* and subsequently subjected to phytochemical characterization and biological evaluation. The bioactive compounds from CH_2_Cl_2_ and EtOAc extracts—maslinic acid (1), corosolic acid (2), eucalyptolic acid (3), quercitrin (5), quercetin (7), and ethyl gallate (8)—were identified as anti-adipogenic constituents of the plant material. *T. loureiri* extract and its active constituents can regulate adipocyte differentiation, suggesting potential mechanistic evidence through which these compounds impact adipogenesis.

## Data Availability

All relevant data supporting the findings of this study are included in the article. Further inquiries can be directed to the corresponding author.

## Supplementary Materials

The following supporting information can be downloaded at: https://www.mdpi.com/article/10.3390/ijms27031374/s1.
