# Supplementary material for: Anti-Adipogenic Effect of Secondary Metabolites Isolated from Tetracera loureiri on 3T3-L1 Adipocytes
Source: Int J Mol Sci. 2026 Jan 29;27(3):1374. doi: 10.3390/ijms27031374 (PMC12898108; doi:10.3390/ijms27031374)
Supplement: Supplementary file 1 [file ijms-27-01374-s001.zip › ijms-4043948-supplementary.pdf]

Supplementary Table S1. ^{1}H and ^{13}C NMR data of compounds 1-10 in CD_3OD.

NO	Compounds 1 and 2			
	**δ_H mult., (*J* Hz)**	**δ_C**	**δ_H mult., (*J* Hz)**	**δ_C**
1		48.2		48.3
2	3.61 (1H, dddd, 11.9, 9.8, 4.9)	69.5	3.62 (1H, dddd, 11.9, 9.8, 4.9)	69.5
3	2.90 (1H, d, 9.8)	84.5	2.91 (1H, d, 9.8)	84.5
4		40.6		40.5
5		56.7		56.7
6		19.6		19.5
7		33.9		34.3
8		40.5		40.8
9		48.9		49.0
10		39.3		39.2
11		24.6		24.5
12	5.25 (1H, t, 3.5)	123.4	5.23 (1H, t, 3.5)	126.6
13		145.4		139.9
14		43.0		29.2
15		28.8		29.2
16		24.1		25.4
17		47.7		49.0
18	2.86 (1H, dd, 14.0, 4.2)	42.8	2.21 (1H, brd, 11.2)	54.5
19		47.3		40.5
20		31.6		40.4

21		34.9		31.9
22		33.8		38.2
23	1.16 (3H, s)	29.3	1.01 (6H, s)	29.3
24	1.00 (3H, s)	17.4	0.81 (6H, s)	17.5
25	0.91 (3H, s)	17.1	1.01 (6H, s)	17.2
26	0.82 (3H, s)	17.8	0.85 (6H, s)	17.6
27	1.01 (3H, s)	26.4	1.12 (6H, s)	24.1
28		181.9		182.1
29	0.81 (3H, s)	33.6	0.89 (3H, d, 7.0)	17.9
30	0.94 (3H, s)	24.0	0.96 (3H, d, 5.6)	21.6

Compound 3					
NO	δ_H mult., (*J* Hz)	δ_C	NO	δ_H mult., (*J* Hz)	δ_C
1		48.5	21		34.9
2	3.84 (1H, m)	67.7	22		33.8
3	4.63 (1H, d, 7.7)	85.6	23	0.90 (3H, s)	29.3
4		40.7	24	0.95 (3H, s)	18.3
5		56.5	25	1.06 (3H, s)	17.1
6		19.5	26	0.84 (3H, s)	17.8
7		33.9	27	1.02 (3H, s)	26.4
8		40.7	28		181.8
9		49.2	29	0.91 (3H, s)	33.5
10		39.3	30	0.94 (3H, s)	24.0
11		24.6	1'		127.9
12	5.26 (1H, m)	123.4	2'	7.20 (1H, d, 2.1)	111.8
13		145.4	3'		149.9
14		43.0	4'		150.5
15		28.8	5'	6.81 (1H, d, 7.7)	116.5
16		24.1	6'	7.08 (1H, dd, 8.4, 2.1)	124.0
17		47.7	7'	7.62 (1H, d, 16.1)	146.5
18	2.87 (1H, dd, 14.0, 4.2)	42.8	8'	6.42 (1H, d, 16.1)	116.2
19		47.3	9'		169.6
20		31.6	-OCH_3	3.90 (3H, s)	56.5

NO	Compound 4	
	**δ_H mult., (*J* Hz)**	**δ_C**
1		123.5
2	7.04 (1H, s)	110.3
3		146.3
4		139.1
5		146.3
6	7.04 (1H, s)	110.3
7		171.4

Compound 5					
**NO**	**δ_H mult., (*J* Hz)**	**δ_C**	**NO**	**δ_H mult., (*J* Hz)**	**δ_C**
2		159.3	3'		146.5
3		136.2	4'		149.9
4		179.6	5'	6.19 (1H, d, 2.1)	116.4
5		163.2	6'	6.19 (1H, d, 2.1)	122.9
6	6.19 (1H, d, 2.1)	100.1	1''	6.19 (1H, d, 2.1)	103.6
7		166.6	2''	6.19 (1H, d, 2.1)	71.9
8	6.36 (1H, d, 2.1)	94.9	3''	6.19 (1H, d, 2.1)	72.2
9		158.6	4''	6.19 (1H, d, 2.1)	73.3
10		105.7	5''		72.0
1'		123.0	6''	6.19 (1H, d, 2.1)	17.6
2'	6.33 (1H, d, 8.4)	117.0			

NO	Compounds 6 and 7			
	**δH mult., (*J* Hz)**	**δC**	**δH mult., (*J* Hz)**	**δC**
2		148.0		148.0
3		137.1		137.2
4		177.4		177.3
5		162.6		162.5
6	6.18 (1H, d, 2.1)	99.3	6.17 (1H, d, 2.1)	99.4
7		165.7		165.9
8	6.40 (1H, d, 2.1)	94.5	6.38 (1H, d, 2.1)	94.5
9		158.3		158.3
10		104.6		104.5
1'		123.8		124.2
2'	8.09 (1H, d, 9.1)	130.7	7.73 (1H, d, 2.1)	116.0
3'	6.91 (1H, d, 9.1)	123.8		146.2
4'		160.6		148.8
5'	6.91 (1H, d, 9.1)	123.8	6.88 (1H, d, 8.4)	116.2
6'	8.09 (1H, d, 9.1)	130.7	7.63 (1H, dd, 8.4, 2.1)	121.7

NO	Compounds 8 and 9			
	**δH mult., (*J* Hz)**	**δC**	**δH mult., (*J* Hz)**	**δC**
1		121.8		127.4
2	7.04 (1H, s)	110.0	7.44 (1H, d, 8.4)	131.0
3		146.5	6.80 (1H, d, 8.4)	116.8
4		139.7		161.1
5		146.5	6.80 (1H, d, 8.4)	116.8
6	7.04 (1H, s)	110.0	7.44 (1H, d, 8.4)	131.0
7		168.6	7.58 (1H, d, 16.1)	146.3
8	4.27 (2H, q, 7.0)	61.6	6.28 (1H, d, 16.1)	115.8
9	1.34 (3H, t, 7.0)	14.6		171.8

NO	Compound 10	
	**δ_H mult., (*J* Hz)**	**δ_C**
2		148.4
5		162.3
6	6.31 (1H, d, 2.1)	98.6
7		167.0
8	6.61 (1H, d, 2.1)	92.8
9		158.2
10		105.4
1'		123.8
2'	8.13 (1H, d, 8.4)	130.8
3'	6.91 (1H, d, 9.1)	116.4
4'		160.8
5'	6.91 (1H, d, 9.1)	116.4
6'	8.13 (1H, d, 8.4)	130.8
$-OCH_3$	3.89 (3H, s)	56.4

Supplementary Table S2. ORO reduce rate (%) of the stained adipocytes.

ORO reduced rate	2.5 μM	5 μM	10 μM	P.C (100 μg/mL)
Maslinic acid	7.16±10.64%	17.43±4.03%	57.39±6.98%	64.30±2.95%
Corosolic acid	−4.57±2.53%	10.68±4.20%	19.35±4.53%	55.80±3.84%
Eucalyptolic acid	7.73±3.88%	32.86±6.48%	75.81±1.75%	61.64±1.88%
Gallic acid	0.85±4.77%	3.19±4.07%	2.75±3.17%	64.33±2.12%
Quercitrin	2.85±1.92%	11.62±2.52%	19.83±5.27%	63.44±3.57%
Kaempferol	−1.04±8.86%	4.74±5.33%	8.22±1.32%	67.34±1.11%
Quercetin	5.19±2.63%	12.51±4.21%	17.61±1.62%	61.81±1.37%
Ethyl gallate	1.65±1.74%	0.80±3.38%	14.66±3.85%	60.81±2.30%
p-coumaric acid	8.70±6.55%	7.83±5.67%	12.11±3.89%	63.60±3.00%
Rhamnocitrin	8.41±4.57%	1.34±6.43%	5.98±4.53%	62.97±1.33%

Supplementary Table S3. Adiponectin and Leptin expression rate (%) of the adipocytes.

Expression	Adiponectin	Leptin
Maslinic acid	86.23±4.73%	54.28±11.79%
Corosolic acid	27.22±6.77%	17.59±0.96%
Eucalyptolic acid	99.59±0.19%	98.74±0.65%
Gallic acid	21.29±7.72%	3.43±1.16%
Quercitrin	8.66±12.83%	12.46±4.45%
Kaempferol	20.90±7.18%	-3.35±3.76%
Quercetin	35.54±6.57%	8.99±3.04%
Ethyl gallate	25.29±7.77%	17.49±3.12%
p-coumaric acid	0.80±10.47%	1.08±4.33%
Rhamnocitrin	20.50±7.57%	7.24±2.71%
P.C (100 μg/mL)	72.97±4.18%	60.05±9.08%
